# A prospective randomized controlled trial to evaluate effect of chewing gum on postoperative ileus in elderly patient after hip fracture

**DOI:** 10.1097/MD.0000000000025321

**Published:** 2021-04-02

**Authors:** Yong-Han Cha, Dae Cheol Nam, Sang-Youn Song, Jun-Il Yoo

**Affiliations:** a Department of Orthopaedic Surgery, Eulji University Hospital, Daejeon; b Department of Orthopaedic Surgery and Institute of Health Sciences, Gyeongsang National University School of Medicine and Gyeongsang National University Hospital, Jinju; c Department of Orthopaedic Surgery, School of Medicine, Gyeongsang National University Hospital, Korea.

**Keywords:** chewing gum, hip fractures, postoperative ileus, prevalence

## Abstract

Factors related to developing postoperative ileus (POI) vary from pharmacologic, inflammatory, hormonal, metabolic, gastrointestinal physiology, neurologic, to psychological factors. Although orthopedic-related incidence of postoperative ileus is about 10%, these studies are limited to spine surgery and pelvic surgery. The purpose of this study was to investigate prevalence of POI and to analyze effect of chewing gum on POI and bowel function in elderly patients after hip fracture surgery.

A prospective randomized controlled trial was conducted at the Gyeongsang National University Hospital. Elderly patients with hip fracture who underwent surgery from March 2017 to June 2018 were eligible to participate. Patients were excluded if they had a mastication disability, impaired cognitive function, previous history of gastrointestinal disease, respiratory disease and low oxygen saturation, hip arthroplasty with causes other than hip fractures, acetabular fractures, periprosthetic fractures, or pathological fractures. Patients with consciousness problem by excessive anesthesia were also excluded. Patients were classified into 2 groups by randomization. Group I received sugar-free gum and were encouraged to chew 6 hours following surgery until the first intestinal gas is released. Group II was given the same postoperative procedure and encouraged to consume water after 6 hours.

After applying exclusion criteria, 74 patients were finally included. Thirty-one patients were classified to Group I and 43 patients were classified to the Group II. Prevalence of POI in all patients with hip fracture was 63.5% (47/74). Prevalence of POI in Group I was statistically significant lower than that in Group II (Group I: 41%, Group II: 79.1%, *P* = .01)

The prevalence of POI in elderly patients with hip fracture was 63.5%. Chewing gum had a significant effect on reduction of POI in elderly patients with hip fractures.

## 1 Introduction

Postoperative ileus (POI) is defined as a temporary problem of gastric and bowel motility after surgery. Symptoms include: painful abdominal distension, inability intake orally, nausea, vomiting, and delay in normal bowel function.^[1,2]^ POI has been reported in approximately 10% to 30% of patients after abdominal surgery and also occurs after obstetric or urological surgery.^[3,4]^ Factors related to developing POI vary from pharmacologic, inflammatory, hormonal, metabolic, gastrointestinal physiology, neurologic, to psychological factors.^[5]^ POI increases hospitalization duration, medical costs, and 30-day readmission rate.^[6,7]^ Complications that may be caused by POI include pulmonary aspiration by nauseous vomiting, dehydration, electrolyte imbalance, and sepsis.^[1]^ Most of these studies, however, are related to non-orthopaedic surgery or drug-specific studies. Although the incidence of postoperative ileus in the orthopedic field is about 10%, these studies are limited to spine surgery and pelvic surgery.^[6,8]^

Elderly patients with hip fracture have high mortality and morbidity rate because of their old age and many underlying diseases.^[9]^ Therefore, the goal for these patients is to reduce complications and preserve function.^[10]^ However, they have multiple risk factors for POI development that can be associated with complications. Older age with reduced gastrointestinal mobility, the use of opioid for pain control, surgical pain and stress, fasting before and after surgery, and effects of anesthetic drugs are likely to cause POI.^[1]^ Moreover, ambulation is limited in these patients. In several studies, patients undergoing gastrointestinal, urologic, obstetric, or gynecological surgery have shown the usefulness of chewing gum for bowel recovery. Chewing gum may serve as a cephalic vagal stimulation of the gastrointestinal tract and be a form of sham feeding, stimulating bowel motility.^[2,11]^ However, in our best knowledge, there are no reports studying the incidence of POI among elderly hip fracture patients and the effects of chewing gum in them who carry higher risks for POI than other surgeries.

Therefore, we hypothesized that chewing gum postoperatively in elderly hip-fracture patients would prevent POI. The purpose of this study was to investigate prevalence of postoperative ileus and to analyze the effect of chewing gum on the reduction of POI in elderly patients with hip fracture.

## 2 Materials and methods

### 2.1 Trial design and participants

**Figure 1:**
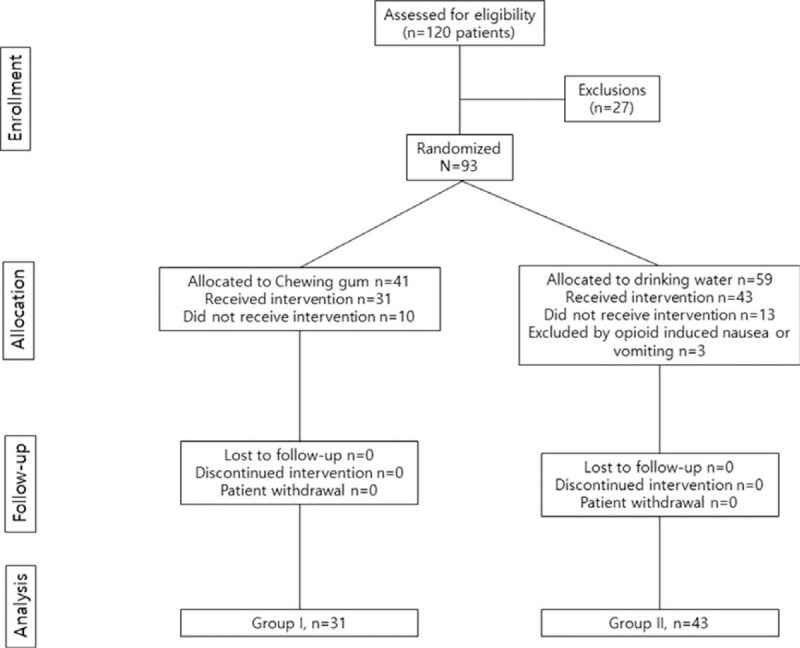
CONSORT diagram.

Total of 120 elderly patients with hip fracture who were diagnosed with unilateral femoral neck or intertrochanteric or subtrochanteric fractures at the Gyeongsang National University Hospital from March, 2017 to June, 2018 were screened for eligibility.(Fig. 1)

The inclusion criteria were acute hip fracture surgery with patients aged 65 or older. The exclusion criteria included the following: 12 Patients with mastication disability (dentures, dental prostheses, etc), 6 patients with impaired cognitive function (obey command step 1 or less, delirium, dementia, Parkinson disease, etc), 2 patients with previous history of gastrointestinal disease, 3 patients with respiratory disease or low oxygen saturation, 2 patients with periprosthetic fractures or pathological fractures, 6 patients with opioid induced nausea or vomiting, 2 patients with refusal to participate. There were no histories of abdominal or pelvic surgery before hip fracture in all patients. Remaining 100 patients were participated in this randomized controlled trial. To randomly divide the patients into 2 groups, sequentially numbered opaque envelopes in which the allocation was sealed were generated by an individual who was not clinically involved in the study. Due to the nature of the study, randomized groups were not blinded as patients, family members and the physician can notice chewing gum.

### 2.2 Interventions

**Figure 2:**
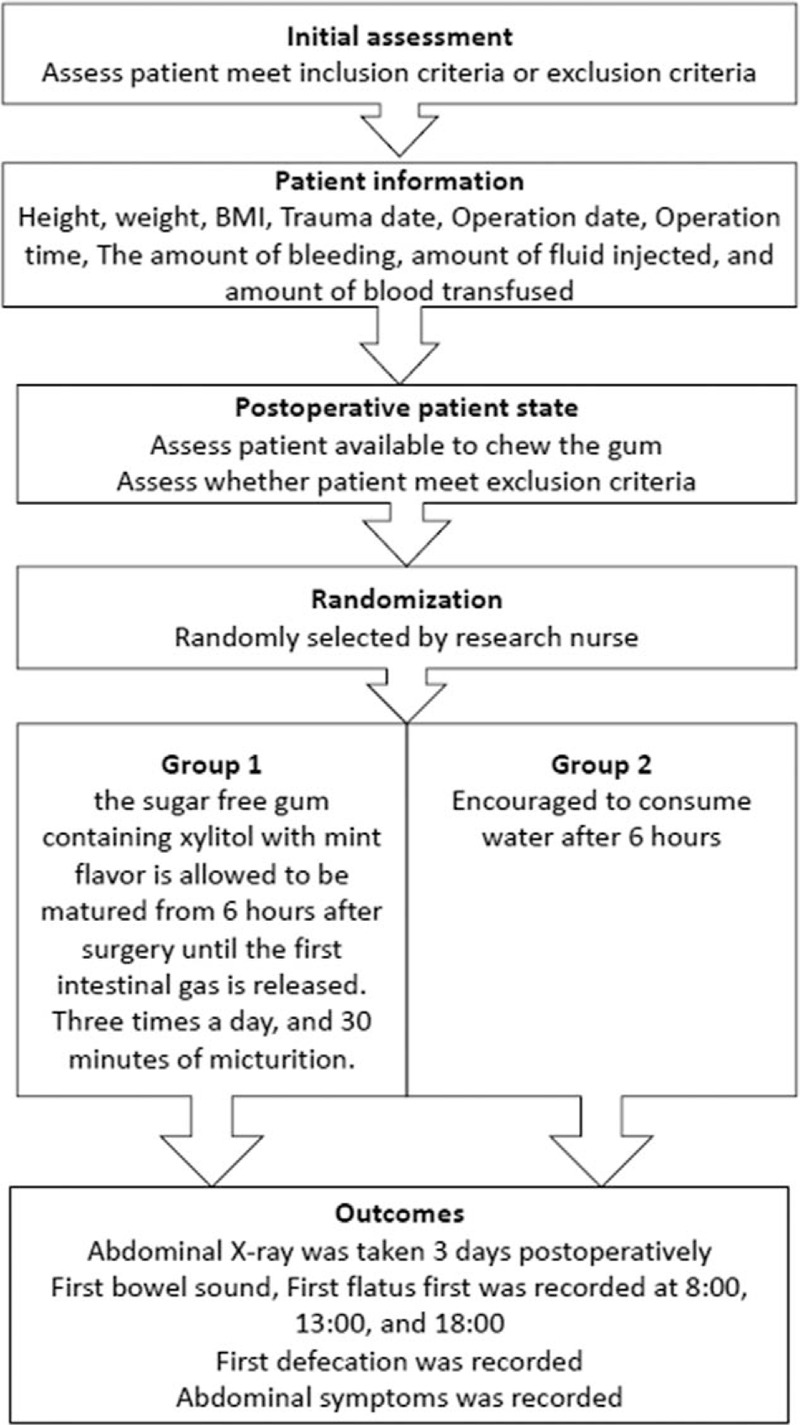
Flow chart of intervention in this study.

Group 1 was required to chew the sugar-free gum (containing xylitol with mint flavor) for approximately 30 minutes each time with an interval of 6 hours from 6th postoperative hour to 4th postoperative day. (Fig. 2)

Group 2 was given the same postoperative procedure and was provided with 200 ml of warm water at 37°C in each time with an interval of 6 hours from 6th postoperative hour to 4th postoperative day.

### 2.3 Perioperative managements

All operations which were scheduled for elective surgery were performed by same surgical team, the same perioperative management, and standardized postoperative care plans. All patients received a uniform anesthesia, which consisted of spinal analgesia with 15 to 30 mg of 0.5% bupivacaine based on weight. Intravenous fentanyl was administered with use of intravenous patient-controlled analgesia (PCA) system (AUTOMED 3300; ACE Medical, Seoul, South Korea) for postoperative pain control. The PCA was set at a bolus of 0.1 mg/kg with a lockout time of fifteen minutes and continuous infusion of 0.1 mg/kg/hour (total regimen, 10 mg/kg/100 ml). The patients were instructed to voluntarily inject the PCA by a button should they need pain control. Breakthrough pain was managed with oral celecoxib 200 mg given twice a day and oral acetaminophen 1 g given every 6 hours.^[12]^ When a patient complained of persistent pain with a VAS pain score of >3, an additional 50 mg of fentanyl was injected intravenously by the duty nurses.^[13]^

Postoperative hemoglobin was checked 48 hours after completion of the operation. Indication for postoperative transfusion of allogenic packed cell was hemoglobin level of less than 8 g/dl or in symptomatic patients. The suction drain was removed until 24 hours after surgery.^[12]^

The oral feeding of both groups started at the 8th postoperative hour with fluids and soft food. They shifted to the normal diet as tolerated.

An abdominal radiograph was taken to investigate POI at 3rd postoperative day for all patients and were confirmed by a radiologist. At 6th postoperative hour, patients were asked whether the first intestinal gas was evacuated. After the first day, patients were reexamined at 8:00, 13:00, and 18:00. The presence of bowel sound was investigated in intervals of 2 hours at each 4 quadrants at 6th postoperative hour, and to shrink the feasible error, the criterion was the presence of 3 bowel sounds in a minute.^[14]^

### 2.4 Definition of postoperative ileus (POI)

POI is diagnosed if 2 or more of the 5 criteria are met on or after day 4 postoperatively without prior resolution of postoperative ileus as following:

1. nausea or vomiting,
2. inability to tolerate an oral diet over the last 24 hours,
3. absence of flatus over the last 24 hours,
4. abdominal distension,
5. radiologic confirmation.^15^

### 2.5 Demographic, operational, and postoperative factors

Demographic factors, operation related factors, and postoperative factors were investigated in all patients. Demographic factors include: age, sex, height, weight, body mass index (BMI), Charlson comorbidity score, preoperative Koval grade, type of fracture.^[15,16]^ Operation related factors were time from trauma to operation, type of surgery, operation time, blood loss, intravenous fluid volume, transfusion volume. The time for sitting up and walking after surgery, and length of hospital stays were also documented for postoperative factors.

### 2.6 Outcomes

Primary outcomes in our study investigated how many elderly patients with hip fracture have the symptoms and signs of POI and how high the prevalence of POI is in these patients. Secondary outcomes gathered the time for first bowel sound, flatus and defecation after hip fracture surgery to evaluate effect of chewing gum on recovery of bowel function.

### 2.7 Sample size calculation

In this study, precision analysis was performed using intraclass correlation coefficients at a target value of 0.8 and a 95% confidence interval set at 0.2, and the minimum sample size was estimated to be 60 patients.

### 2.8 Statistical methods

We used the Chi-Squared or Fisher exact tests for categorical variables and the independent *t* test for numerical variables. All two-sided *P* values <.05 were considered significant. Statistical analyses were conducted using SPSS v 22.0 (SPSS, Inc., Chicago, IL).

## 3 Results

Among the 100 patients who participated in the study, 41 patients were randomly assigned to chewing gum whereas 59 were allocated to the control group. Of these 100 patients, 10 patients in the chewing group and 16 patients in the control group did not meet the inclusion criteria in this study. Finally, 31 subjects in the study group and 43 subjects in the control group were enrolled in the study. No significant differences were showed in demographic factors between groups (Table 1).

**Table 1:** Demographic characteristics of included patients.

Variable	Group I (N = 31)	Group II (N = 43)	*P* value
Age (years ± SD)	75.29 ± 11.29	78.51 ± 12.47	.734
Sex (male/female)	15/16	25/18	.481
Height (m ± SD)	1.62 ± 0.08	1.58 ± 0.08	.163
Weight (kg ± SD)	60.70 ± 11.81	57.12 ± 9.91	.390
BMI (kg/m^2^)	23.01 ± 3.20	22.06 ± 4.67	.672
CCI	1.94 ± 2.11	2.21 ± 1.98	.575
Koval grade			.109
4 ≤	5 (16.1%)	15 (34.9%)	
≤ 3	26 (85.9%)	43 (65.1%)	
Femoral fracture type			.731
Neck	10 (32.3%)	15 (34.9%)	
Intertrochanter	20 (64.5%)	25 (58.1%)	
Subtrochanter	1 (3.2%)	3 (7.0%)	

BMI = body mass index, CCI = Charlson‘s Comorbidity score, N = number, SD = standard deviation.

The operation time of Group I was longer than that of Group II (Table 2). And the amount of bleeding and infused fluid was higher in Group I, but there was no statistical difference in the amount of blood transfusion between 2 groups. The time to sitting up after surgery was 9.87 hours [standard deviation (SD) ± 1.63] in Group I and 9.95 (SD ± 1.64) in Group II (*P* = .837) (*P* = .837). And, the time to postoperative ambulation was 2.71 days (SD ± 0.69) in Group I and 2.76 days (SD ± 0.73) in Group II (*P* = .785). There was no statistical significant difference in length of hospital stay between the 2 groups (Group I: 15.09 ± 0.83 days, Group II: 15.07 ± 0.91, *P* = .910). There was no inhospital mortality in both groups.

**Table 2:** Operation related factors of included patients.

Variable	Group I (N = 31)	Group II (N = 43)	*P* value
Trauma to operation duration (days ± SD)	3.95 ± 4.71	14.36 ± 21.84	.037
Type of surgery			.419
Internal fixation	25 (80.6%)	30 (69.7%)	
Arthroplasty	6 (19.4%)	13 (30.3%)	
Operation time (hrs ± SD)	162.00 ± 109.30	89.26 ± 36.11	.010
Blood loss (ml ± SD)	747.50 ± 830.89	530.00 ± 465.03	.015
Intravenous fluid volume (ml ± SD)	1153.00 ± 493.67	797.50 ± 247.15	.022
Transfusion volume (ml ± SD)	690.00 ± 798.61	798.61 ± 528.09	.103

hrs = hours, N = number, SD = standard deviation.

Prevalence of POI in the study was 63.5%. For prevalence of POI in relation to chewing gum, Group I showed 41.9% (13/31) and Group II showed 79.1% (34/43) (*P* = .001). Abdominal symptoms such as nausea, vomiting, or abdominal pain occurred in 12 patients in Group I and 29 patients in Group II (*P* < .05). There was no statistically significant difference between the 2 groups in time for first bowel sound, flatus and defecation after hip fracture surgery (*P* < .05).(Table 3)

**Table 3:** Postoperative factors of included patients.

Variable	Group I (N = 31)	Group II (N = 43)	*P* value
Time for sitting up after surgery (hrs ± SD)	9.87 ± 1.63	9.95 ± 1.64	.837
Time for walking after surgery (day ± SD)	2.71 ± 0.69	2.76 ± 0.73	.785
Postoperative ileus (N %)	13 (41.9%)	34 (79.1%)	.001
Time for first bowel sound after surgery (hrs ± SD)	6.39 ± 1.09	6.51 ± 1.16	.638
Time for first flatus after surgery interval (hrs ± SD)	15.32 ± 9.73	13.12 ± 9.63	.337
Time for first defecation after surgery interval (hrs ± SD)	50.77 ± 35.50	74.95 ± 113.49	.166
Length of hospital stay (days ± SD)	15.09 ± 0.83	15.07 ± 0.91	.910

hrs = hours, N = number, SD = standard deviation.

## 4 Discussion

The principal findings of this study were that the prevalence of postoperative ileus (POI) in elderly patients with hip fracture was 63.5% and prevalence of POI in the group chewing gum was significantly lower than that of the control group (41.9% in Group I vs 79.1% in Group II, *P* = .001. Although POI is a physiological arrest of gastrointestinal function due to surgical stress, there was no statistically significant difference between the 2 groups in time for first bowel sound, flatus and defecation after hip fracture surgery.

The increase in human life span caused an increase in the proportion of the elderly population and the number of surgeries performed in them also increased.^[17]^ Patients in this age group have high postoperative complication rates due to multicomorbidity and old age itself, and various efforts are required to decrease their high mortality and complications.^[10]^ Postoperative complications of hip fracture patients vary, including pneumonia, urinary tract infection, and cholecystitis, but information on postoperative ileus is insufficient.^[18,19]^ There are several studies on POI after orthopedic surgery, but not from hip fracture surgery. Al Maaieh MA et al,^[20]^ performed a retrospective cohort study using 596 patients after lateral lumbar interbody fusion to assess the independent risk factors of POI after the procedure. They reported the incidence of POI as 7%. In addition, independent risk factors for POI included a history of gastroesophageal reflux disease, posterior instrumentation, and lateral lumbar interbody fusion at Lumbar 1 to 2. Parvizi et al^[21]^ evaluated the incidence and risk factors for POI after total joint arthroplasty using regional anesthesia and multimodal pain management protocols. They reported that POI is a common postoperative complication following total joint arthroplasty, occurring in 0.7% (31/4567) of patients. The ileus was treated successfully in 29 patients during the hospitalization. One patient died from this complication, and another 1 required sigmoid colon resection because of perforation. The risk factors for developing POI after total joint arthroplasty were older age, male sex, hip arthroplasty, and prior history of abdominal surgery. They reported that the type and dose of narcotic medications did not appear to influence the development of POI. Lee et al^[22]^ reported incidence of POI in 612 orthopedic surgeries. They found that 2.1% experienced POI. Comparing to previous studies, incidence of POI in our study is very high. We believe that this is because hip fracture patients tend to be older and carry higher comorbidity risks than those in other studies. Aging deteriorates organ function and induce physiologic change.^[23]^ Thus, they have inadequate functional reserve for the stress of surgery or medication. The most effective method to prevent POI is known to be early ambulation, but these patients are limited from doing so. Our findings suggest that POI care is very important in elderly hip fracture patients.

In several studies, intervention for the prevention of POI have included appropriate intravenous fluid resuscitation, correction of electrolyte imbalance, early ambulation, prokinetic motility drugs, nicotine, and chewing gum, etc.^[24]^ There have been reports that these interventions reduce gut edema and inflammation. In particular, chewing gum has been reported to stimulate bowel movement and flatus passage and to promote defecation by cephalic-vagal reflex.^[25]^ Some studies also reported that chewing gum is a very cost-effective intervention for restoring intestinal function.^[3,4,12]^ In our study, intervention group (group chewing gum) showed less prevalence of POI than the control group. Even in the high old age and low physiological activity patient with hip fracture, we found that it was effective in preventing POI.

Since abdominal or gynecological surgeries are performed with direct incision in the intestine or indirectly in contact with the bowel, mechanical factors such as adhesion and bowel obstruction appear to be important among the various factors that affect the occurrence of POI. Therefore, it may be necessary to use various drugs to prevent POI in these patients.^[26]^ However, POI occurring in elderly patients with hip fracture is mainly due to low physiological activity, psychological stress, and ambulation problem, with mechanical factors playing a less significant role compared to abdomino-gynecological surgeries. This is because the prevalence of POI in the intervention group was higher than that of control group in our study, but there was no statistically significant difference between the 2 groups terms of time to first bowel sound, flatus and defecation after hip fracture surgery. Thus, although many drugs are effective in preventing POI, many elderly patients are limited from taking additional drugs due to medications for underlying comorbidity. Therefore, considering cost effectiveness, chewing gum is considered an effective POI prevention method in elderly patients with hip fracture.^[3,4,12]^

There are several limitations of this study. First, the size of the patient group was small compared to other studies, and the patients excluded were relatively high. Second, selection bias may be present because of the exclusion of patients at risk for aspiration pneumonia. Third, we could not evaluate the bowel status and history of analgesia use before the operation on the patients. Nevertheless, we collected data prospectively, and imaging and clinical findings were all used for diagnosis of POI. In addition, many factors influencing the occurrence of POI were well controlled after admission, such as termination of preoperative analgesics, same postoperative protocol for pain control.

## 5 Conclusions

The prevalence of postoperative ileus in elderly patients with hip fracture was 63.5%. Chewing gum had a significant effect on reduction of postoperative ileus in elderly patients with hip fractures.

## Author contributions

**Conceptualization:** Yong-Han Cha, Jun-Il Yoo.

**Data curation:** Dae Cheol Nam, Jun-Il Yoo.

**Formal analysis:** Yong-Han Cha, Dae Cheol Nam, Jun-Il Yoo.

**Investigation:** Yong-Han Cha.

**Supervision:** Dae Cheol Nam, Sang-Youn Song.

**Validation:** Sang-Youn Song.

**Writing – original draft:** Yong-Han Cha, Sang-Youn Song, Jun-Il Yoo.
